# Epiglottis reshaping using CO_2 _laser: A minimally invasive technique and its potent applications

**DOI:** 10.1186/1746-160X-4-15

**Published:** 2008-07-25

**Authors:** Constantinos Bourolias, Jiannis Hajiioannou, Emil Sobol, George Velegrakis, Emmanuel Helidonis

**Affiliations:** 1 Department of Otolaryngology, University of Crete School of Medicine, Heraklion, Crete, Greece; 2 Biophotonics Laboratory, Institute on Laser and Information Technologies, Russian Academy of Sciences, Troitsk, Russia

## Abstract

Laryngomalacia (LRM), is the most common laryngeal abnormality of the newborn, caused by a long curled epiglottis, which prolapses posteriorly. Epiglottis prolapse during inspiration (acquired laryngomalacia) is an unusual cause of airway obstruction and a rare cause of obstructive sleep apnea syndrome (OSAS).

We present a minimally invasive technique where epiglottis on cadaveric larynx specimens was treated with CO2 laser. The cartilage reshaping effect induced by laser irradiation was capable of exposing the glottis opening widely. This technique could be used in selected cases of LRM and OSAS due to epiglottis prolapse as an alternative, less morbid approach.

## Introduction

Cartilage reshaping techniques are used in the field of reconstructive surgery in cases of congenital or acquired cartilage anomalies. One approach to reshape native cartilage tissue relies on rapid photothermal heating to accelerate stress relaxation of cartilage [1].

Laryngomalacia (LRM) is the most common congenital laryngeal anomaly of the newborn, commonly caused by the in-drawing of the supraglottis with inward curling of both sides of the epiglottis, or by the backward projection of an extremely lax epiglottis [2]. Obstructive sleep apnoea syndrome (OSAS) is caused by obstruction or narrowing of the airway at various levels. Epiglottis prolapse during inspiration (acquired laryngomalacia) is an unusual cause of airway obstruction and a rare cause of OSAS [3].

We propose a minimally invasive technique for the treatment of selected cases of LRM and OSAS due to epiglottis prolapse.

## Technique

For the purpose of epiglottis reshaping, CO_2 _laser irradiation was employed on larynx specimens acquired from three patients suffering of larynx cancer treated with total laryngectomy. There was no spread of the disease at any surface of the epiglottis. The laser beam was delivered on the lingual surface of the epiglottis, by means of a Sharplan 1040 CO^2 ^laser at a wavelength of 15600 nm. The laser beam was focused to the desired spot size with a barium fluoride lens, transparent at 15600 nm, with a focal length of 400 mm, mounted on a surgical microscope. Intermittent exposures were used (pulse repetition rate 1 Hz), the spot diameter was 2 mm, the exposure time was 0.5 second, and the output power was 3 W. In each exposure the achieved energy was 48 J × cm^-2^. Twenty to 30 pulses of 0.5 second or 60 to 90 J were required to remodel the epiglottis. These laser parameters were based on the results of our previous experiments [4].

## Results and Discussion

**Figure 1:**
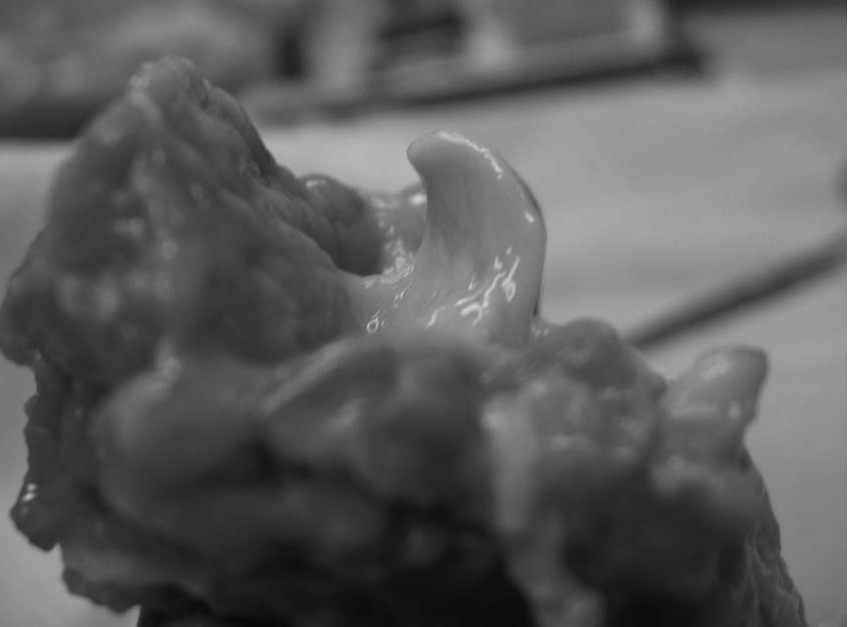
**Epiglottis before laser irradiation.** (Cadaveric specimen).

**Figure 2:**
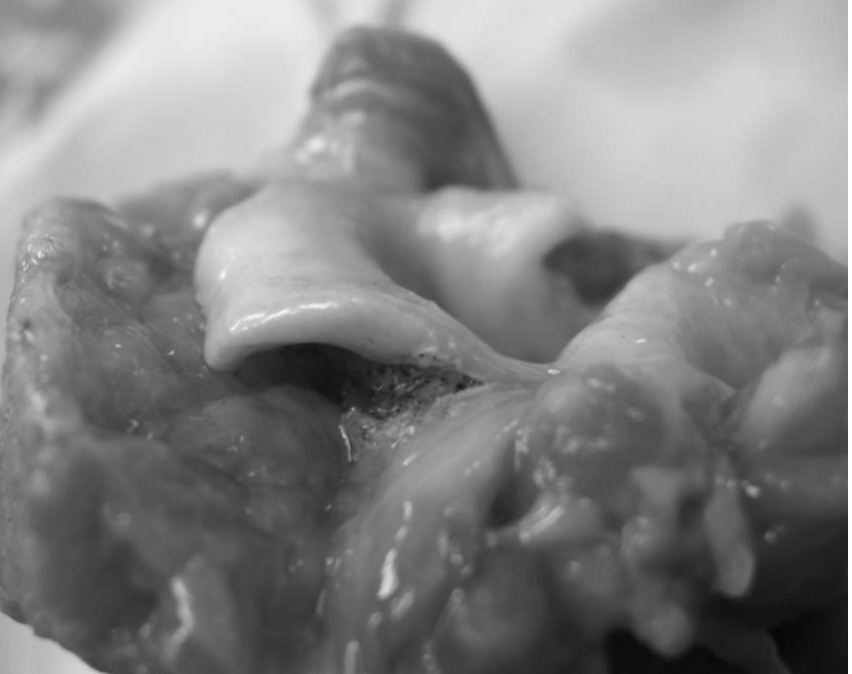
**Epiglottis after laser irradiation**. The epiglottis has acquired a new curved shape warping towards the direction of laser beam application. (Cadaveric specimen).

**Figure 3:**
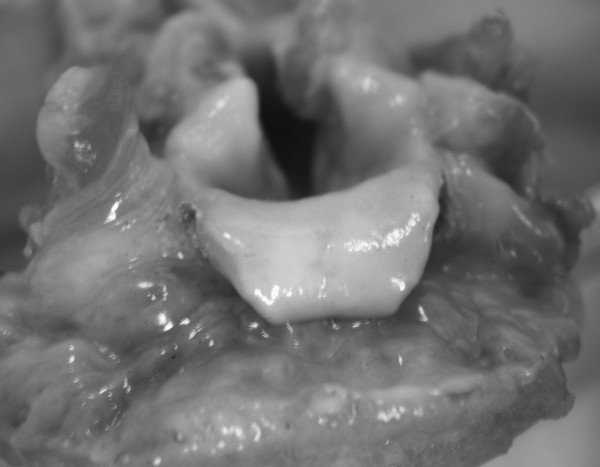
**Epiglottis after laser irradiation (Superior frontal view)**. The glottis opening is widely exposed. (Cadaveric specimen).

The epiglottis reshaping effects follows mucosal coagulation by the laser irradiation as the beam is applied to the superficial cartilage layers. The shape of the epiglottis before and after irradiation is shown in Figures 1, 2 and 3. As it was expected, based on previous published experiments on cartilage tissue [5], the epiglottis acquired a new curved shape warping towards the direction of laser beam application thus exposing widely the glottis opening. Although these experiments have been initially performed in cadaveric tissues with different behavior compared to the living ones we believe that the subsequent scar formation, which is expected to occur during the healing process, would further retract the epiglottis anteriorly.

Cartilage reshaping techniques are used in the field of reconstructive surgery in cases of congenital or acquired cartilage anomalies. One approach to reshape native cartilage tissue relies on rapid photothermal heating to accelerate stress relaxation of cartilage grafts [1]. Laser mediated cartilage reshaping (thermochondroplasty) is a non-ablative, low-intensity interaction where heat accelerates stress relaxation in deformed cartilage specimens resulting in shape change. Cartilage can be reshaped when heated to approximately 60–75°C [1] Different types of lasers have been employed (CO2, Er: Glass, Holmium etc) with similar results for the treatment of nasal septal deviations [4,5] and protruding ears [6].

LRM is the most common congenital laryngeal anomaly, the most frequent cause of stridor in children and generally a benign, self-limited process [2]. The term LRM was first used by Jackson and Jackson in 1942 to designate stridor caused by the indrawing of the supraglottis with inward curling of both sides of the epiglottis, or by the backward projection of an extremely lax epiglottis [7]. The exact pathophysiology of LRM remains obscure. It is however established that it leads to a dynamic supraglottic collapse in inspiration. Three anatomical abnormalities have been chiefly implicated: short arytoepiglottic folds, a long curled epiglottis which prolapses posteriorly and the presence of bulky arytenoids with loose mucosa, which prolapses forward on inspiration. These features may be seen in combination or as separate entities [8]. LRM has been reported to contribute to adult with OSAS, although the incidence is low [9].

OSAS is caused by obstruction or narrowing of the airway at various levels. OSAS and LRM are two different entities. Occasionally, they may have a common etiology (an elongated, flaccid, and lax epiglottis), that is displaced posteriorly during inspiration causing airway obstruction (acquired laryngomalacia) [3]. Moreover a correlation between the shape of the epiglottis and OSAS has been reported [10].

A variety of surgical procedures have been proposed to manage severe LRM. Common procedures are aryepiglottoplasty in combination with glossoepiglottopexy with 'cold' instruments while currently the most popular performed techniques is supraglottoplasty with the CO_2 _laser [2]. Supraglottoplasty refers to any surgical procedure in which supraglottic laryngeal tissues are excised. The procedures in this series are subdivided according to the region. These include the lingual mucosal surface of the epiglottis (epiglottopexy), the posterior edges of the epiglottis (epiglottoplasty), and the mucosa of the aryepiglottic fold (aryepiglottoplasty) and the suprarytenoidal mucosa (arytenoidoplasty) [2]. Moreover epiglottidectomy has been advocated for the treatment of OSAS due to epiglottis prolapse [2].

Recently these techniques have been modified by use of endoscopic instruments thus becoming less invasive with benefits in terms of decreased morbidity and improvement of quality of life [8,11,12].

The above-mentioned techniques have been proved effective but are not free of complications. The most common described in the literature for these procedures include bleeding, infection, edema, aspiration, dysphagia, supraglottic stenosis, synechia formation, respiratory distress, and death [2]. Many of them are attributed to the excessive removal of laryngeal tissue [2].

In cases of LRM and OSAS where the main etiologic factor is the inward curling or the backward projection of an extremely lax epiglottis is obvious that a less invasive technique might be equally effective in alleviating the symptoms evading serious complications. Laser thermochondroplasty is cartilage-reshaping technique, which provides accuracy in obtaining the desired shape of the irradiated cartilage while minimizing adjacent tissue damage in the same time. Following the laser application the cartilage shape remains constant and the tissue fully functioning [5].

It has to be emphasized that these reported results on cadaveric specimens are preliminary and further research in vivo is required. Currently a series of experiments is being conducted on animals (canines) in an effort to determine the efficacy of this method on living specimens using different types of laser (CO2, Er: glass) as well as the healing process and possible complications.

The next step is to design a clinical trial in selected patients suffering of OSAS and newborns with LRM comparing preoperative and postoperative polysomnogrpaphy, for establishing the effectiveness of this technique in these pathologic entities.

We believe that in selected cases of LRM and OSAS due to epiglottis malformations such as inward curling or backward projection of an extremely lax epiglottis, laser epiglottoplasty could be proven a safe, less morbid alternative approach; however further data are necessary to support this hypothesis.

## Authors' contributions

CB participated in the sequence alignment. JH participated in the design of the study and performed the statistical analysis. EM carried out the molecular genetic studies, participated in the sequence alignment and drafted the manuscript. GV participated in the sequence alignment. EH conceived of the study, and participated in its design and coordination. All authors read and approved the final manuscript.
